# Role of N-Terminal Amino Acids in the Potency of Anthrax Lethal Factor

**DOI:** 10.1371/journal.pone.0003130

**Published:** 2008-09-03

**Authors:** Pradeep K. Gupta, Mahtab Moayeri, Devorah Crown, Rasem J. Fattah, Stephen H. Leppla

**Affiliations:** Laboratory of Bacterial Diseases, National Institute of Allergy and Infectious Diseases, National Institutes of Health, Bethesda, Maryland, United States of America; University of Helsinki, Finland

## Abstract

Anthrax lethal factor (LF) is a Zn^+2^-dependent metalloprotease that cleaves several MAPK kinases and is responsible for the lethality of anthrax lethal toxin (LT). We observed that a recombinant LF (LF-HMA) which differs from wild type LF (LF-A) by the addition of two residues (His-Met) to the native Ala (A) terminus as a result of cloning manipulations has 3-fold lower potency toward cultured cells and experimental animals. We hypothesized that the “N-end rule”, which relates the half-life of proteins in cells to the identity of their N-terminal residue, might be operative in the case of LF, so that the N-terminal residue of LF would determine the cytosolic stability and thereby the potency of LF. Mutational studies that replaced the native N-terminal residue of LF with known N-end rule stabilizing or destabilizing residues confirmed that the N-terminal residue plays a significant role in determining the potency of LT for cultured cells and experimental animals. The fact that a commercially-available LF preparation (LF-HMA) that is widely used in basic research studies and for evaluation of vaccines and therapeutics is 3-fold less potent than native LF (LF-A) should be considered when comparing published studies and in the design of future experiments.

## Introduction

Anthrax is a disease caused by spore-forming, Gram-positive bacterium, *Bacillus anthracis*. The virulence of *B. anthracis* depends on production of two major virulence factors-the gamma-linked poly-D-glutamic acid capsule and anthrax toxin. The toxin is composed of protective antigen (PA) and two catalytic moieties, lethal factor (LF) and edema factor (EF) [1], [2]. The binary combination of PA and LF is known as lethal toxin (LT), while PA and EF together constitute edema toxin (ET). PA is the central receptor-binding component which delivers LF and EF into the cytosol of mammalian cells. EF is a calmodulin-dependent adenylate cyclase [3] while LF is a Zn^+2^-dependent metalloprotease that cleaves several MAPK kinases [4], [5].

Injection of LT induces death of experimental animals, which can occur in 1–2 days in mice or as little as 38 minutes in rats. LT has been found to have many physiological and pathological effects [6], [7], including but not limited to impairing endothelial barrier function [8] and glucocorticoid receptor activity [9], and inducing necrosis or apoptosis in macrophages [10], [11]. LT induces a shock-like vascular collapse similar to that observed in anthrax-infected animals and humans. Because LT plays a key role in virulence during anthrax infections, substantial effort has been directed to the development of vaccines and therapeutics that target this toxin. These efforts depend on the availability of reliable, economical sources of purified toxin components having consistent and well characterized properties. Various expression hosts have been used for the purification of PA and LF, including *Escherichia coli*, *Bacillus subtilis*, and *B. anthracis* [12]–[14]. This laboratory originally produced native PA, LF, and EF from the avirulent *B. anthracis* Sterne strain [15], [16] and later developed systems for efficient expression and purification of PA and LF as recombinant molecules from avirulent strains of *B. anthracis* [17], [18]. In the course of these studies we noted differences in LF potency between sources and individual preparations which suggested that variability at the N-terminus of the protein might have an impact on potency. This was consistent with prior studies showing that the stability of LF fusion proteins in the cytosol of cells depended on identity of the N-terminal residue [19] pointing to the involvement of the N-end rule in determining susceptibility to ubiquitinylation and subsequent degradation by the proteasome [20].

The N-end rule of protein degradation relates the *in vivo* stability of a protein to the identity of its N-terminal residue. Ubiquitin (Ub) ligases target protein substrates that bear specific (destabilizing) N-terminal residues [20], [21]. The corresponding degradation signal called the N-degron consists of a protein substrate's destabilizing N-terminal residue and an internal Lys residue, the latter being the site of attachment for a poly-Ub chain. A ubiquitylated substrate is then targeted to and degraded by the proteasomes [22]. It is of interest to note that unlike many bacterial toxins that have a strong bias of Arg over Lys [23], LF has a substantial number of Lys in the N-terminal region that are potential ubiquitination sites. A truncated LF protein, LF_1–254_ (also designated LF_n_), containing the PA binding domain of LF, was shown to follow the N-end rule in cells [19], since addition of destabilizing residues at its N-terminus (e.g., Arg) increased the protein's degradation rate in reticulocyte lysate and cells. LF produced by *B. anthracis* contains the sequence AGGH, which becomes the N-terminus of the mature protein following cleavage of its signal peptide by the bacterial signal peptidase. In our studies we initially noted that the presence of two additional N-terminal residues in recombinant LF decreased its potency approximately 3-fold. This prompted a systematic study of the role of the N-terminus, which is reported here.

## Methods

### Cell culture and cytotoxicity assays

RAW264.7 cells were used for cytotoxicity studies. Cells were grown in Dulbecco's modified Eagle medium containing 2 mM Glutamax, 25 mM HEPES and 50 µg/ml gentamicin supplemented with 10% fetal calf serum (all from Invitrogen, Carlsbad, CA) at 37°C in 5% CO_2_. For cytotoxicity assays, cells were plated in 96-well plates 24 h prior to use. Cells were treated with varying concentrations of LF in the presence of a fixed concentration of PA (250 ng/ml) for 3–4 h. Cell viability was assessed by the addition of MTT [3-(4,5-dimethylthiazo-2-yl-02,5-diphenyltetrazolium bromide] (Sigma, St. Louis, MO) at a final concentration of 0.5 mg/ml, incubation for another 45 min at 37°C, and release of the blue pigment produced by viable cells using 0.5% (w/v) sodium dodecyl sulfate (SDS), 25 mM HCl in 90% (v/v) isopropanol. A microplate reader was used to measure the A_570_ to quantify cell survival.

### Plasmids and mutagenesis

Plasmid pSJ115 [18] was used to express wild type and mutated LF proteins. The Quick Change-II site directed mutagenesis kit (Stratagene, La Jolla, CA) was used for manipulations in pSJ115 according to the manufacturer's instructions. Mutations were confirmed by DNA sequencing, and for each construct the entire gene was also sequenced to confirm that there were no other mutations present.

### Production and purification of proteins

Proteins PA, FP59, and all LF variants were produced and purified from *B. anthracis* strain BH450 as described earlier [18], [24] . Samples of LF used for comparison were purchased from List Biological Laboratories (Campbell, CA).

### Processing of LF proteins by factor Xa

Factor Xa (Novagen, Madison, WI) was used to cleave off the Myc tag from purified, mutated LF proteins. In a typical small scale digestion, 25 µg of protein was incubated at room temperature with 0.3 units of factor Xa in 1 X reaction buffer containing 50 mM Tris-HCl, pH 8.0, 100 mM NaCl, 5 mM CaCl_2_. After 14 h incubation at 4°C, 2 mM EDTA and 1 mM DTT was added. Large scale preparations used the same conditions. N-terminal sequencing was performed to determine the N-terminus of LF proteins.

### LF cellular affinity determinations

The affinity of LF proteins to cell-bound PA was measured by Schild plot analyses [24], [25]. These studies used CHO WTP4 cells, which are not killed by LF, and the cytotoxic fusion protein FP59 [26], [27], in which the N-terminus of LF is fused to the ADP-ribosylating domain of *Pseudomonas* exotoxin A. CHO cells were maintained in alpha minimal essential medium supplemented with 5% fetal bovine serum, 2 mM glutamine, 50 µg of gentamicin/ml, and 25 mM HEPES. In Schild plot analyses, cells were incubated with various concentrations of FP59 plus PA (constant at 12 nM) in the presence of different fixed concentrations of the LF protein being analyzed, which acted as a non-toxic competitor of FP59. After 3 h, the toxins were replaced by media containing 10 mM NH_4_Cl to inhibit further toxin delivery to the cytosol [24], [28], [29]. Cells were then incubated for 48 h at 37°C followed by MTT addition for cell viability determination. Data were analyzed by Graphpad Prism software to calculate binding constants.

### Animal experiments

Female Fischer 344 rats (Taconic Farms Germantown, NY, 170–190 g) were injected via the tail vein (200 µl/rat) with a mixture of PA+LF (LT), prepared in sterile PBS. Concentrations and doses of LT refer to the amounts of each component (i.e. 10 µg LT is 10 µg PA+10 µg LF and 100 µg LT is 100 µg PA+100 µg LF). The same PA preparation was used in combination with different LF proteins. Rats were observed continuously for signs of malaise and mortality. Balb/cJ mice (Jackson Labs, Bar Harbor, ME) were injected IP (1 ml/mouse) with different doses of LT and monitored for up to 7 days for malaise and mortality. All animal experiments were performed under protocols approved by the NIAID Animal Care and Use Committee.

## Results

### Toxicities of LF proteins produced from avirulent *B. anthracis* strains

**Figure 1:**
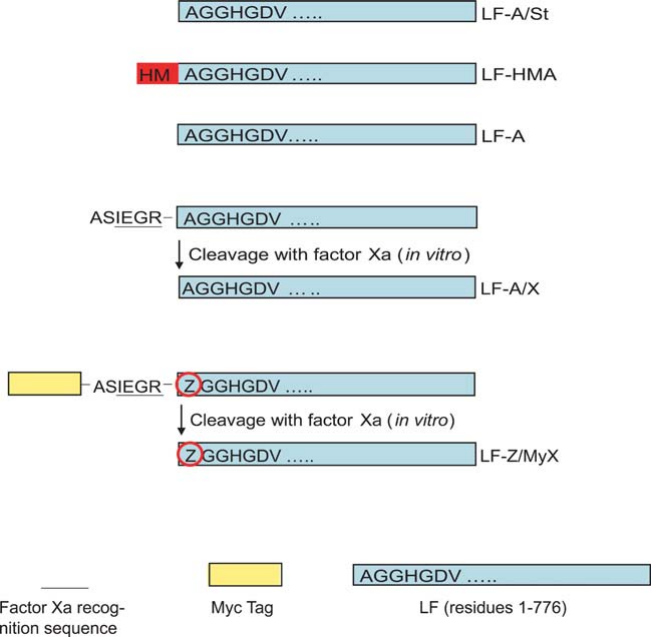
Schematic representation of various LF proteins. All proteins (except LF-A/St which is wild type LF produced from Sterne strain) were generated as secreted proteins from *B. anthracis* BH450. All proteins contained a signal peptide that is cleaved by signal peptidases during secretion. Constructs with a factor Xa recognition sequence alone or preceded by the Myc epitope tag are labeled “/X” or “/MyX”, respectively. These proteins were produced as precursor proteins followed by cleavage with factor Xa protease to generate the indicated N-termini. Residue (Z) circled in red indicates the residue mutated for each protein, i.e., Z = A, H, M, R, F, G in different mutated LF constructs.

LF has been expressed and purified in many laboratories from a variety of expression hosts, including *E. coli*, *B. subtilis* and *B. anthracis*. This laboratory produces LF from non-toxigenic strains of *B. anthracis*. Originally, we prepared LF as well as PA and EF from the Sterne strain of *B. anthracis* [15], [16] grown in a manner similar to that used to produce the currently licensed anthrax vaccine [30]. A sample of LF produced in this way, kept frozen at −80°C since 1984, and here designated LF-A/St, was available for use in the current study (Fig. 1). Subsequently, to facilitate production of mutated proteins and to eliminate concerns about contamination of one toxin component with the others, the LF gene was cloned into a recombinant shuttle vector, pSJ115, which was transformed into various *B. anthracis* strains cured of the pXO1 and pXO2 virulence plasmids [18]. LF expressed from pSJ115 is here termed LF-HMA to denote the presence of the two residues His-Met (HM) added at its N-terminus due to the cloning manipulations. This expression system is licensed to List Biological Laboratories (Campbell, CA), and the LF sold by them is therefore also LF-HMA. All the LF proteins produced in our lab from *B. anthracis* are secreted proteins containing signal peptides that are cleaved by the endogenous signal peptidases during secretion.

Over the course of several years, we noted that multiple batches of LF-HMA were less toxic than LF-A/St (data not shown). Based on the hypothesis that the reduced toxicity of LF-HMA was due to the two additional residues (HM), we mutated pSJ115 to remove the two codons specifying HM, producing LF-A, with the native N-terminal sequence matching LF-A/St (Fig. 1). Because secreted proteins can undergo degradation by co-secreted bacillus proteases in the bacterial culture supernatant or during protein purification, we also constructed LF proteins with cleavable N-terminal sequences or tags. Thus, the LF-A/X protein (Fig. 1) was obtained from a secreted precursor protein processed by the bacterial signal peptidases to yield a LF protein having a 6-residue N-terminal extension. The purified protein was then cleaved with the factor Xa protease to yield an N-terminal Ala (Fig. 1). A similar construct was made in which the factor Xa cleavage site is preceded by a Myc tag, which can be used for affinity purification and for detection (LF-A/MyX, Fig. 1). The ability to precisely control the N-terminus of each protein by factor Xa cleavage also eliminated any heterogeneity that might occur if the selection of cleavage site by the signal peptidase was inexact. We found that the insertion of the Myc tag and factor Xa recognition sequences after the signal peptide had no measurable impact on protein yields (data not shown). Precursor proteins purified from supernatants of *B. anthracis* BH450 were cleaved with factor Xa to remove the N-terminal extensions and to obtain mature mutated LF proteins with different N-terminal amino acids (Fig. 1). Western blot studies with anti-Myc antibodies were used to verify the initial presence of the Myc tags and their subsequent removal by cleavage with factor Xa (data not shown).

**Figure 2:**
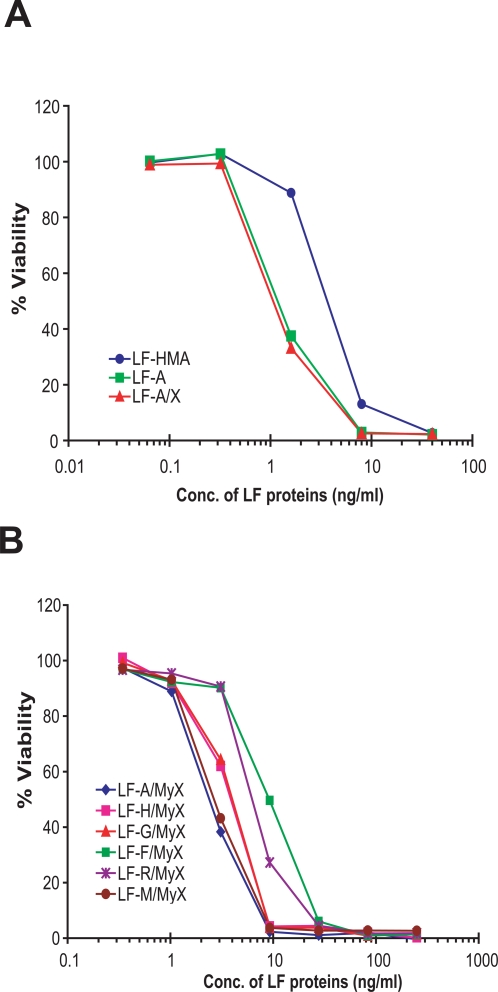
Cytotoxicity of LF proteins to RAW264.7 cells. RAW264.7 cells were incubated with various concentrations of LF-HMA, LF-A, and LF-A/X proteins (A) or LF proteins with mutated N-termini (B) and a fixed concentration of PA (250 ng/ml). Cell viability was assessed at 3 h. Percent viability was calculated relative to cells treated with medium (no toxin).

LF-A and LF-A/X showed enhanced toxicity as compared to LF-HMA in macrophage cytotoxicity assays (Fig. 2A, Table 1). The EC_50_ values (concentration required to kill 50% of the cells) of LF-A and LF-A/X were approximately 3-fold lower than those of LF-HMA (Fig. 2A, Table 1), and were comparable to the values found in other experiments for LF-A/St (data not shown). Because LF-A/St, LF-A and LF-A/X have the exact same N-terminal sequence while LF-HMA has two additional residues at the N-terminus (Fig. 1) but the latter has lower potency, we performed more extensive analyses of the role of the N-terminal residue, described below.

**Table 1:** Toxicity of LF proteins to RAW 264.7 cells.

LF Proteins	EC_50_ (ng/ml) *	Source of data	Estimated half life (h)#		
			Reticulocyte lysates	Vero-Dr22 cells	Yeast
LF-HMA	3.7	Fig. 2A	3.5	1.08	0.16
LF-A	1.2	Fig. 2A	4.4	2.9	>20
LF-A/X	1.1	Fig. 2A	4.4	2.9	>20
LF-A/MyX	1.4	Fig. 2B	4.4	2.9	>20
LF-R/MyX	6.1	Fig. 2B	1	0.83	0.03
LF-G/MyX	4.0	Fig. 2B	30	9.8	>20
LF-F/MyX	9.5	Fig. 2B	1.1	0.51	0.05
LF-H/MyX	3.9	Fig. 2B	3.5	1.08	0.16
LF-M/MyX	1.5	Fig. 2B	30	19.3	>20

* EC_50_ is the (effective) concentration of toxin required to kill 50% of cells. RAW264.7 cells were incubated with LF proteins and 250 ng/ml PA and viability determined as described in Methods.

# Estimated half life is that measured for a Beta-galactosidase test protein having the same N-terminus when incubated in rabbit reticulocyte lysate or when produced *in vivo* in *Saccharomyces cerevisiae* [31]. Half life in Vero-Dr22 cells mentioned here is for diphtheria toxin as reported earlier [32].

### The N-end rule is predictive of LF potency

The “N-end rule” states that for certain proteins, the identity of the N-terminal amino acid residue determines the rate of cytosolic ubiquitinylation and thereby the rate of degradation by the proteasomes. The fact that the His at the N-terminus of the LF-HMA is classified as a destabilizing residue compared to Ala, at least in *Saccharomyces cerevisiae* [31] and in Vero-Dr22 cells [32] , suggested that the N-end rule might apply to LF stability in cells. To examine this possibility, the N-terminus of LF was substituted with the amino acid residues Gly, Arg, Met, Phe, and His (Fig. 1). These proteins and LF with the native N-terminal Ala were produced with the Myc tag and factor Xa site, and designated as LF-Z/MyX (Fig. 1, with Z representing the substitution and X denoting presense of a factor Xa cleavage site). According to the N-end rule, Gly and Met are classified as stabilizing residues while Arg and Phe are considered destabilizing ones [22], [31], [33].

Cytotoxicity assays in the mouse macrophage RAW264.7 cell line were performed to compare the activities of the mutated LF proteins. LF with the most stabilizing residue, Met (LF-M/MyX) was as toxic as wild type sequence LF-A/MyX but more toxic than LF with destabilizing residues Phe (LF-F/MyX) and Arg (LF-R/MyX) (Fig. 2B). LF proteins with His (LF-H/MyX) or Gly (LF-G/MyX) at their N-terminus were less toxic than LF-M/Myx and LF-A/MyX but more toxic than LF-F/MyX and LF-R/MyX (Table 1). The destabilizing residue Phe clearly had a large impact on toxin activity, as the EC_50_ was increased ∼6-fold compared to LF with the most stabilizing residue, Met (Table 1). These data clearly support the hypothesis that the N-end rule applies to LF potency in cells, likely by controlling cytosolic stability of the toxin.

### *In vivo* role of N-terminal residue of LF

The rapid lethality that occurs in Fischer rats following intravenous injection of LT provides an assay that is widely used in the evaluation of LF-directed therapeutics. When injections are done accurately, the response is highly predictable, with little deviation in time to death (TTD) for a given dose. Historically, this laboratory's TTD following injection of 10 µg LT using various preparations of LF-HMA fell in a range of 73–110 min. We found that the same dose of LT using LF-A resulted in an average TTD of 63 min (Table 2). Furthermore, LF constructs with the N-end rule stabilizing residues Met or Gly, and the intermediate stabilizing residues, His and Ala, resulted in a similarly low TTD (Table 2). LF constructs having the N-end rule destabilizing residues Arg and Phe, however, showed higher average TTD of 78 min and 122, respectively (Table 2). The injection of a high dose of 100 µg LT/rat did not reduce the TTD for LF-HMA preparations below 50 min (Table 2). A 100 µg dose of LF-A, however, resulted in a TTD of 38–39 min in every rat. LF-A/X, produced by cleavage with factor Xa, also resulted in a similar rapid 37 min TTD in every rat. It was gratifying that these results exactly match, to the minute, the theoretical minimum TTD derived by extrapolation in studies done in 1984 [34] with a LF preparation equivalent (and possibly identical) to the LF-A/St used in these studies.

**Table 2:** Toxicity of LF proteins in rats.

LF Proteins	Dose (µg/rat)	Survival*	TTD (min)	Average TTD (min)
LF-HMA	10	0/25	97, 97, 102, 130, 91, 92, 93, 93, 93, 93, 94, 94, 95, 73, 75, 95, 95, 96, 96, 97, 102, 102, 106, 107, 109	97
LF-A	10	0/25	62, 70, 53, 55, 56, 57, 57, 61, 61, 62, 65, 65, 66, 71, 76, 60, 60, 60, 55, 58, 59, 71, 73, 75, 76	63
LF-R/MyX	10	0/3	80, 77, 76	78
LF-F/MyX	10	0/3	98, 145, 124	122
LF-G/MyX	10	0/3	66, 65, 66	66
LF-M/MyX	10	0/3	60, 65, 65	63
LF-H/MyX	10	0/3	62, 65, 65	64
LF-A/MyX	10	0/3	62, 63, 61	62
LF-HMA	100	0/3	51, 51, 50, 54, 53	52
LF-A	100	0/3	39, 39, 39, 38, 38, 38, 38, 39	39
LF-A/X	100	0/6	37, 37, 37	37

* Fisher 344 Rats were injected with 10 µg PA or 100 µg PA plus equivalent amount of each LF preparation (IV) and monitored for minutes to death.

**Figure 3:**
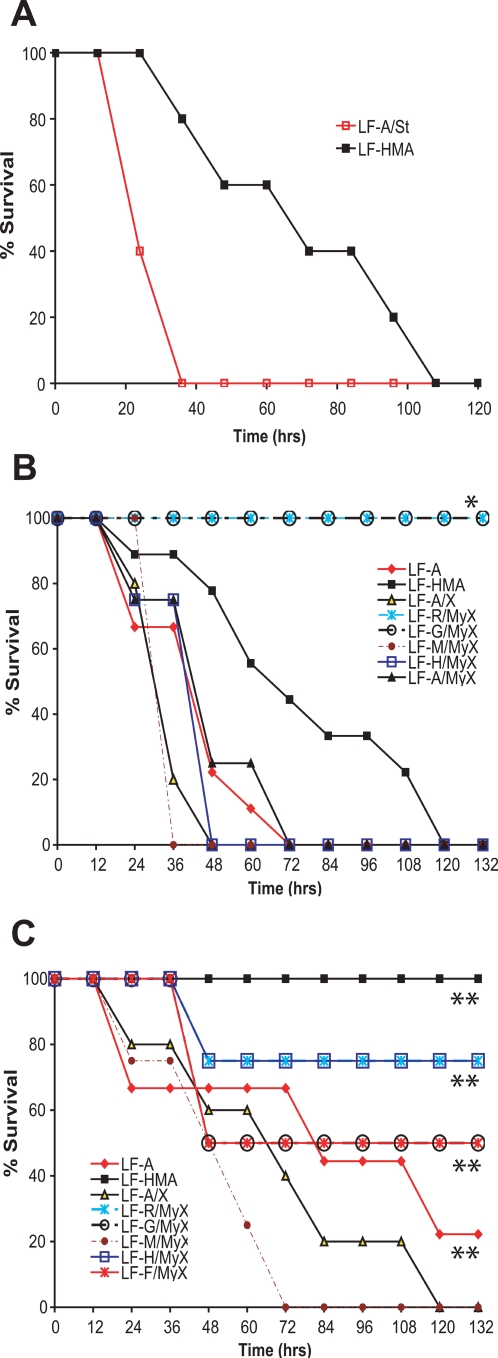
Toxicity of LF proteins to mice. (A) LF-HMA and LF-A/St (100 µg) were injected in Balb/cJ mice in combination with 100 µg PA via the IP route and survival was monitored for 120 h. Each group contained n = 5 mice. (B) LF proteins (100 µg) were injected in Balb/cJ mice in combination with 100 µg PA via the IP route and survival was monitored for 120 h. Mouse numbers used in this experiment were as follows: LF-HMA (n = 9), LF-A (n = 9), LF-A/X (n = 5), LF-G/MyX (n = 3) and all other groups n = 4. (*) The animals in LF-G/MyX and LF-R/MyX groups exhibited substantial malaise starting at 24 h and throughout the experiment and were euthanized at 128 h to prevent suffering in accordance with approved animal protocols. (C) LF preparations (40 µg) were injected in Balb/cJ mice in combination with 40 µg PA via the IP route and survival was monitored for 120 h. Mouse numbers used in this experiment were as follows: LF-HMA (n = 14), LF-A (n = 9), LF-A/X (n = 5) and all other groups n = 4. (**) The surviving animals in these groups did not display any signs of malaise over the last 48 h of the experiment.

This laboratory has also used the mouse lethality test extensively. We found that LF-A/St, the material prepared in 1984, was significantly more potent than LF-HMA (Fig. 3A). We extended these studies and tested LF-A, LF-A/X, LF-R/MyX, LF-F/MyX, LF-G/MyX, LF-M-MyX and LF-H/Myx in Balb/cJ mice at two doses, 100 and 40 µg injected IP (Fig. 3B and 3C). At the 100 µg dose, LF-HMA was less potent than all tested LF preparations except LF-G/MyX and LF-R/MyX. These two proteins resulted in extended malaise in mice, with no toxin-induced animal deaths (LF-F/MyX was not tested in this study). All other LF preparations had significantly lower TTD than LF-HMA. At the 40 µg dose the LF-M/Myx was clearly the most potent construct, being the only preparation resulting in 100% lethality at this low dose. Thus the results from the *in vivo* mouse studies, despite having higher variability than the rat studies, did support the hypothesis of involvement of N-end based stability of proteins in animal toxicity.

### Affinity of LF proteins for cell-bound PA

**Figure 4:**
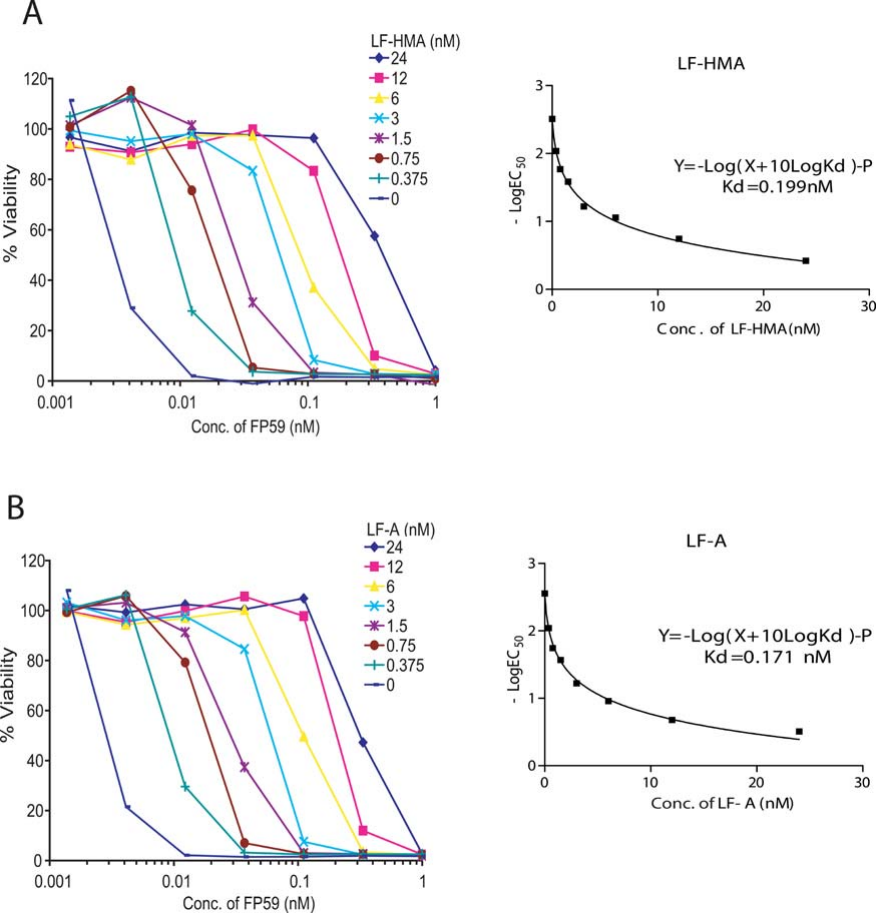
Evaluation of the apparent binding affinities of LF-HMA and LF-A for PA using Schild Plot analyses. CHO WTP4 cells were incubated with various concentrations of FP59 plus a set concentration of PA (12 nM) and different concentrations of LF-HMA (A) or LF-A (B) for 3 h. After toxin removal, cells were incubated with the toxin-free medium containing 10 mM NH_4_Cl for 48 h before assessment of cell viability. Schild Plot analyses were performed as described in “Methods” to assess *K*_d_s of each LF protein for PA. Inserts shown in panels A and B are non-linear regression curves obtained from these analyses using GraphPad Prism.

To determine whether the difference in the activity of LF-HMA and LF-A is caused by differences in binding affinity, we measured the apparent affinities (*K*_d_) of LF-HMA and LF-A for protective antigen (PA). For these studies, CHO WTP4 cells which are anthrax LT-resistant but sensitive to FP59 (a chimeric toxin of LFn and catalytic sub-unit of *Pseudomonas* exotoxin A) were used. FP59 is highly toxic to CHO WTP4 cells due to its catalytic activity i.e. ADP-ribosylation of elongation factor-2 and blocking of protein synthesis [27]. Binding affinity for different LF-HMA and LF-A proteins was compared by measuring the sensitivity of CHO WTP4 cells to PA plus FP59 when LF-HMA or LF-A were used as competing inhibitors of toxicity. Addition of fixed concentrations of LF-HMA or LF-A shifted the PA plus FP59 cytotoxicity dose-response curves (Fig. 4). The EC_50_ values of FP59 plus PA cytotoxicity dose responses in the presence of various fixed concentrations of LF-HMA or LF-A were determined and a non-linear regression fit analysis was performed using the equation Y = −log(X+10log *K*_d_)−P, where Y = −log(EC_50_) (nM), X = [LF-HMA] or [LF-A] (nM), and P is a constant, (see http://www.graphpad.com/curvefit/schild.htm for details). The results show that the apparent affinities of LF-HMA and LF-A to PA are very similar, 0.19 nM and 0.17 nM, respectively (Fig. 4). Thus, these data show that the difference in the activity of LF-HMA and LF-A is not due to the altered binding to PA.

## Discussion

The production of LF (and PA) from *B. anthracis* has a number of advantages that, together with the improvements reported here, recommend its continue use as a host for production of LF and PA, which have value as biological research reagents and as components of anthrax vaccines. Secretion to the culture supernatant from the native host organism assures that processing (e.g., by the signal peptidase) and folding will be optimal. Because *B. anthracis* secretes few proteins and little protease activity, due to truncation and inactivation of the global transcriptional regulator PlcR [35], [36], the secreted toxin proteins typically constitute more than half of the supernatant proteins. In this laboratory, a 3-L shake flask culture typically yields 200 mg of purified LF. A final advantage when the LF is to be used in studies of signal transduction pathways is the absence of gram-negative endotoxin.

Data collected over several years in our laboratory indicated that purified LF-HMA, either made in our laboratory or purchased from List Laboratories, was less toxic in macrophage toxicity assays when compared to LF purified from the *B. anthracis* Sterne strain. LF-HMA differed from LF-A/St only in two N-terminal residues (His and Met). We hypothesized that the decreased potency of LF-HMA could be due to the lower stability of protein once it is internalized to the cell cytosol, as might occur if the N-end rule were operative for LF. Previous studies had provided evidence that the N-end rule applied to proteins containing the N-terminal LF domain [19], and since recognition of proteins by the ubiquitinating enzymes typically depends on a small region near the N-terminus of proteins, it was expected that the N-end rule would also apply to native, full-size LF. We tested this hypothesis by constructing recombinant LF proteins having the same sequence as LF-A/St, either as a result of signal peptide cleavage, or by cleavage of precursor proteins at a factor Xa protease site (with or without a Myc tag upstream). All three such proteins, LF-A, LF-A/X and LF-A/MyX, had three-fold lower EC_50_ values (i.e., higher potencies) than LF-HMA in macrophage toxicity assays, similar to what was historically observed for LF-A/St. In the Fischer rat assay of LT potency, the lowest TTD observed for LF-HMA was 50 min. In contrast, LF-A and LF-A/X were lethal to rats within 37–39 min, respectively, in exact correspondence to a theoretical minimum TTD previously obtained by extrapolation in 1984 [34].

We further tested the role of N-end rule in controlling the LF stability in cells by producing mutant LF proteins with different N-termini. The N-end rule predicts that proteins bearing charged basic (type 1, e.g. Arg or Lys) or large hydrophobic (type 2, e.g. Phe or Trp) N-termini are targeted more rapidly to the proteasomes. These residues are termed “destabilizing” residues. On the other hand, certain N-terminal residues such as Met or Gly are highly stabilizing [31]. The N-end rule has been observed for many proteins which have shown similar but not identical patterns in terms of the effect of a specific amino acid on the protein stability [32], [33], [37], [38]. In our studies, LF with the stabilizing residue Met was the most toxic in both cell culture and two animal models. As predicted by the N-end rule, the LF protein with the stabilizing Gly N-terminus was comparable to a LF construct with native wild type amino terminus (Ala) in cell toxicity studies and the rat lethality model. Addition of the potent destabilizing residue, Phe, to LF resulted in significantly decreased LF potency for both macrophages and rats, while an intermediate destabilizing residue, Arg, showed predictable intermediate effects both *in vitro* and *in vivo*. The amino acid His has been shown to have variable destabilizing effects for different proteins depending on the cell type [31], [32]. In the case of LF, both His and the native N-terminal residue, Ala, did not have a destabilizing effect and behaved similarly to the LF construct with the stabilizing Gly residue, however, they behaved differently in cell toxicity experiments. The results of these mutational studies, especially those observed for the highly stabilizing residue Met (in LF-M/MyX) and the highly destabilizing residue Phe (in LF-F/MyX) clearly indicate that the N-terminus has a significant impact on the activity of lethal factor both in cell toxicity studies, as well as in animals.

Although our data implicates the N-end rule as determining and explaining the relative potencies of the LF proteins discussed here, we cannot exclude that other factors may contribute to the effects we observed. The N-terminal domain of LF initiates the entrance of LF into the channel formed by the PA heptamer, but residues near the N-terminus are not involved in binding to PA [39], [40], nor is there evidence that they are needed for translocation. Thus, N-terminal truncation of LF by more than 13 residues was needed to strongly decrease the ability of LF to enter the PA heptamer channel [41], suggesting that the identity of individual residues near the N-terminus is not critical in translocation. This makes it less likely that the substantial effects we see from substitution of single N-terminal residues on activity are due to alternation of the translocation process.

The clear role of LF stability in the Fischer rat model of LT sensitivity is especially striking. The variability in TTD and potency of the different LF proteins tested in this study can easily explain the high degree of variability seen in the dosages required by different laboratories for LT lethality in various animal models. LF preparations purified from bacterial hosts can yield a heterogeneous population of proteins with a range of N-termini (data not shown). The mixture of different N-termini in a particular LF preparation can greatly affect the potency. In fact, in our laboratory, where LF production is a routine and frequently performed procedure, we have on rare occasions obtained preparations of LF that produce much higher TTD in the uniquely sensitive Fischer rat model when compared to previously validated standard LF preparations. Similarly, different potencies have also been noted with commercially available LF preparations (data not shown). We believe that variable levels of different N-termini in each preparation, generated through the purification process or by bacterial proteases may explain potency differences in these and other laboratories' preparations. We suggest that new approaches to LF preparation, such as generation of uniform N-end termini as demonstrated in this work, offers a route to limiting this variability.
